# Co‐production in sleep research: A scoping review of current practices and future directions

**DOI:** 10.1111/jsr.14476

**Published:** 2025-02-09

**Authors:** Emma Louise Gale, Raahat Manrai, Lorna Caddick, Aja Murray, Heather C. Whalley, Daniel Smith, Maria Gardani

**Affiliations:** ^1^ Division of Psychiatry, Centre for Clinical Brain Sciences University of Edinburgh Edinburgh UK; ^2^ Population and Behavioural Science, School of Medicine University of St Andrews St Andrews UK; ^3^ School of Philosophy, Psychology and Language Sciences University of Edinburgh Edinburgh UK; ^4^ Generation Scotland, Centre for Medical Informatics University of Edinburgh Edinburgh UK; ^5^ School of Health in Social Science University of Edinburgh Edinburgh UK

**Keywords:** design and development, engagement, insomnia, intervention, participatory action research, public and participant involvement

## Abstract

Sleep is essential for mental and physical health, and research in the field has substantially expanded over the past 50 years. Co‐production methodology has been increasingly used within health and social care research, and refers to collaboration between researchers, policy makers, community partners and wider stakeholders. The aim of this scoping review was to detail the use of co‐production methods within sleep research. A review of the existing literature was conducted using seven databases following PRISMA‐ScR guidelines. Search terms included objective and subjective sleep outcomes, and the use of co‐production research methodologies. Sixteen studies were included in the final review: 10 studies used solely qualitative co‐production methods to inform intervention design and development (sleep as a primary outcome [*n* = 5] and as a secondary outcome [*n* = 5]), and six studies used co‐production methodologies to establish sleep as a priority outcome for future research. Most studies used consultation approaches to design interventions (*n* = 8), instead of using co‐design teams (*n* = 2). Two studies focusing on intervention development recruiting participants from clinical populations with poor sleep, other studies recruited from those with other underlying conditions or a healthy population. The most common limitations of the included studies were small sample size, researcher driven topics/domains for the PAR components, under‐representative samples and COVID‐19 pressures. Future sleep research should consider the use of co‐production methodologies from the study conceptualisation, through to the design, development and implementation of research to further benefit the intended research population.

## 1 INTRODUCTION

Sleep is essential for mental and physical health, and research in the field has substantially expanded over the past 50 years (Hassan et al., 2021; Perez‐Pozuelo et al., 2020). There is growing public interest in sleep within health research (Lemoine et al., 2022; Lim et al., 2023; Ramar et al., 2021). Sleep disturbances are considered to be a primary or secondary symptom of various mental health disorders (Freeman et al., 2020). Sleep disruption has a further impact on cognitive processes like memory, attention and verbal skills (Calhoun et al., 2020; Shaw et al., 2022). Research has demonstrated that co‐production methodologies offer significant benefits across various health fields, including mental health and other areas, by fostering more inclusive and effective interventions (Jakobsson et al., 2023). Applying these principles to sleep research could similarly enhance the relevance and impact of interventions by ensuring they are more closely aligned with the needs and experiences of diverse populations. Additionally, integrating co‐production methodologies may improve our understanding of sleep health disparities across different groups (Dregan & Armstrong, 2011; Jehan et al., 2018), and support the development of tailored interventions that are both accessible and acceptable to those who need them most.

### 1.1 Co‐production methodology

Co‐production refers to the process of collaboration between wider community partners, stakeholders and researchers (Vargas et al., 2022). Co‐production as a research tool has been increasingly used and recognised as an important methodology within different stages of health and social care research (Albert et al., 2023). While co‐production broadly falls under the participatory action research (PAR) framework (Pettican et al., 2023), there are different terminologies used to refer to this type of research method, namely PPI/E (Patient‐Public Involvement/Engagement), Community Based Participatory Research (CBPR) or PAR (Pettican et al., 2023). This review will use the term “co‐production” to highlight the dynamic process of co‐creation and co‐design in different components of sleep research. Co‐production involves collaboration toward a shared objective, with all stakeholders actively contributing their skills, knowledge and experience (Smith et al., 2023). Furthermore, co‐production is a transdisciplinary method used in evaluation, policy making, service development and provision along with conventional research (Beresford & Beresford, 2021; Pettican et al., 2023).

Co‐production became a prevalent research method within health and social care research after the INVOLVE project (INVOLVE, 2020) funded by the National Institute for Health Research (NIHR; Coldham, 2021). This type of research process is an ethical imperative that involves engaging, respecting and including multiple perspectives who provide context‐specific lived experience and practical knowledge to scientific research (Coldham, 2021). Despite these guidelines, studies often adopted some advisory consultations or PPI, and there are missing links with the co‐production cycle, for example, data collection, data analysis and the use of co‐researchers to implement the study (Galende‐Sánchez & Sorman, 2021). Co‐production involves engaging stakeholders with lived experience from the very beginning of the research project, incorporating their insights and expertise on a specific topic (Smith et al., 2023). They actively participate as co‐creators of resources and study designs, and also serve as co‐researchers throughout the process, contributing to the delivery, data collection and dissemination of research findings (Smith et al., 2023). Cowdell et al. identified the use of top‐down and bottom‐up strategies for the co‐production of research with individuals possessing specific lived experiences, ensuring their experiential knowledge directly informs the research framework and outcomes (2022). The National Institute for Health and Care Excellence (NICE) also provides evidence‐based guidelines to implement co‐production within intervention‐based research (Causer et al., 2024).

### 1.2 Co‐production within sleep research

As previously mentioned, co‐production as a research methodology is gaining emphasis within different disciplines, especially mental health research. In the context of sleep research, co‐production has the potential to play a crucial role in several areas, including informing research questions, shaping recruitment and retention strategies, translating findings into practice, and developing relevant measures. However, it appears that within sleep research, co‐production has been applied more narrowly, with a primary focus on measurement. This limited scope highlights an opportunity to broaden its application and fully integrate stakeholders into all stages of the research process.

Sleep, often measured in combination with mental health measures, has successfully incorporated different tools like digital methods and low‐intensity technology to objectively measure sleep over time, but has been slower in implementing co‐production methods within research designs. A review by Arnardottir et al. recommended future sleep research to co‐design tools of measurement with patients and healthcare professionals to improve diagnosis and treatments (2021). While polysomnography (PSG) remains the gold‐standard for sleep assessment for some sleep disorders, the invasive and costly nature of this method has led to new assessment protocols (Chen et al., 2023). At‐home solutions are becoming more popular, such as type 2 PSG (ambulatory; Withers et al., 2022), actigraphy devices (Smith et al., 2018), wearables (de Gans et al., 2024; Scott et al., 2020), nearables (Yoon & Choi, 2023) and app‐based subjective measures (Fino & Mazzetti, 2019). Collecting sleep data can be demanding, and so participant views and demographics will influence factors such as the acceptability of the assessment technique, duration of data collection and wording of instructions (Stephanie et al., 2020).

### 1.3 Present scoping review

We aimed to synthesise existing literature on co‐production methodologies used in sleep research. It aims to understand how co‐production or the overall PAR has been developed within contemporary sleep research.

### 1.4 Characterisation of co‐production within the review

As stated above, co‐production involves researchers and community partners to co‐design and co‐create knowledge within the context of a research paradigm (von Köppen et al., 2022). The primary focus of this review was to describe the current use of co‐production methodology within sleep research. We aimed to highlight where co‐production has been conducted during the research process. In a scoping review by Cowdell et al., the authors have used different phrases such as “consultation”, “co‐design”, “co‐creation”, “participatory research design” and “co‐production” to describe patient/participant involvement within the research design and process (Cowdell et al., 2022). This review makes use of the term co‐production for all the above to highlight the collective engagement of researchers and participants within a research study.

## 2 METHODOLOGY

### 2.1 Literature search

A systematic search of the following seven databases took place in April 2024: MEDLINE, PsychInfo, EMBASE, CINAHL, Scopus, Global health, and Allied and Complimentary Medicine (AMED). The search was conducted following the guidelines outlined in the 2020 update of the Preferred Reporting Items for Systematic Reviews and Meta‐Analyses (PRISMA) extension for Scoping Reviews (Tricco et al., 2018). The search terms are shown in Table 1. The search was limited to original peer‐reviewed studies, English language (minimum: title) and human studies. There were no limitations on the publication timeframe, or the geographic location of the studies included. This scoping review was registered on OSF prior to undertaking (https://osf.io/bmv6zhttps://osf.io/bmv6z).

**TABLE 1:** Search terms used.

Line number	Search term
1	Chronotype OR circadian* OR social jetlag OR sleep* OR (biological adj4 rhythm) OR clock* OR evening* OR morning* (Title/Abstract filter)
2	Co‐prod* OR co prod* OR PPI* OR participant involvement OR participant engagement OR participatory action OR lived experience* OR peer research OR advisory group* (Title/Abstract filter)
3	Systematic* OR meta* (Title only)
	1 AND 2 NOT 3

### 2.2 Selection of studies

Inclusion and exclusion criteria were used to select studies for this review. Initially, duplicates were eliminated, and the remaining articles were evaluated first by title and abstract, and then by full text, adhering to the predetermined inclusion and exclusion criteria (Table 2). One author conducted all database searches (EG), and two authors independently screened the citations at title and abstract level (EG: 100% of citations; RM: 33% of citations) and full text level (EG and RM: 100% of citations). Final decisions on study inclusion or exclusion were made by consensus.

**TABLE 2:** Inclusion and exclusion criteria.

	Inclusion	Exclusion
	Search terms 1 and 2	
Population	General public (children and adults), clinical populations	–
Concept	Studies with a co‐production element in the methodology, including (but not limited to) participant involvement and engagement, PAR, advisory groups, peer research	No co‐production elements within the methodology Qualitative and/or quantitative analysis only Incl. observational, prospective, longitudinal, cohort, cross‐sectional, nested, intervention, trial, clinical study, randomised controlled trials study designs
Context	Sleep was assessed as a primary, secondary or exploratory outcome	No clear information on how sleep was included in the co‐production aspect of the study
Types of evidence source	English language (minimum: abstract), published, peer‐reviewed, human studies across all population groups	Descriptive reviews, articles, case reports, letters, abstract only, conference abstracts, unpublished work, systematic reviews, meta‐analyses and summary studies

Abbreviations: PAR, participatory action research.

### 2.3 Inclusion and exclusion of studies

Studies were included if a co‐production element was incorporated in the methodology, including (but not limited to) participant involvement and engagement, PAR, advisory groups and peer research, and where the study assessed subjective or objective sleep as an outcome.

### 2.4 Narrative synthesis of studies

Included studies were grouped by the co‐production methodology used, by the stage of the study co‐production method (such as conceptualisation, development, design, data collection methods and dissemination) and by the type of sleep research being used. It is important to note that focus groups, workshops and advisory groups have been categorised separately within the review to reflect their phrasing in the respective studies.

## 3 RESULTS

### 3.1 Data extraction

**FIGURE 1:**
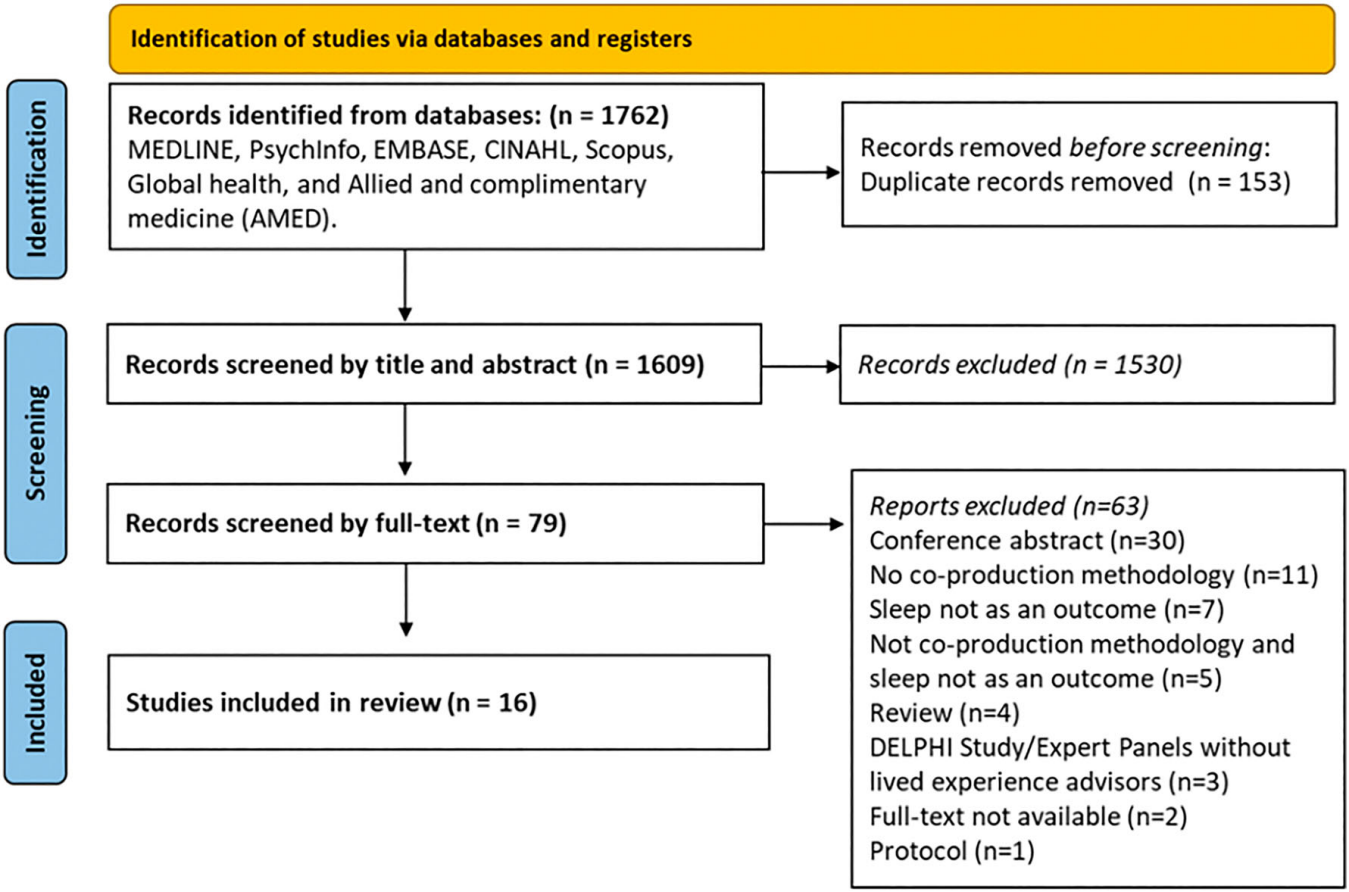
PRISMA‐Scr flow chart (Tricco et al., 2018).

A total of 1762 records were identified from the systematic search, 153 duplicate records were removed, leaving 1609 records to be screened by title and abstract. After screening by title and abstract, 79 records were included for screening at full‐text level. Overall, 16 studies met the inclusion criteria for this scoping review (Alamoudi et al., 2024; Andemeskel et al., 2023; Bedson et al., 2019; Brown et al., 2024; Caswell et al., 2020; Cherniack et al., 2019; Crudgington et al., 2020; Dewa et al., 2019; Garbers et al., 2023; McConachie et al., 2018; Namazi et al., 2023; Perkes et al., 2022; Rapaport et al., 2018; Ryan et al., 2023; Vandendriessche et al., 2023; Vera San Juan et al., 2022; Figure 1).

### 3.2 Study characteristics

Included studies were conducted in the UK (Alamoudi et al., 2024; Bedson et al., 2019; Brown et al., 2024; Caswell et al., 2020; Crudgington et al., 2020; Dewa et al., 2019; McConachie et al., 2018; Rapaport et al., 2018; Vera San Juan et al., 2022; *n* = 9), USA (Andemeskel et al., 2023; Cherniack et al., 2019; Garbers et al., 2023; Namazi et al., 2023; Ryan et al., 2023; *n* = 5), Australia (Perkes et al., 2022; *n* = 1), and Belgium and The Netherlands (Vandendriessche et al., 2023; *n* = 1; Table 3). Though there was no restriction on the date of publication, all included studies were published between 2018 and 2024, with a reduction in studies over the period of COVID‐19 that increased directly after: 2024 (Alamoudi et al., 2024; Brown et al., 2024; *n* = 2); 2023 (Andemeskel et al., 2023; Garbers et al., 2023; Namazi et al., 2023; Ryan et al., 2023; Vandendriessche et al., 2023; *n* = 5); 2022 (Perkes et al., 2022; Vera San Juan et al., 2022; *n* = 2); 2020 (Caswell et al., 2020; Crudgington et al., 2020; *n* = 2); 2019 (Bedson et al., 2019; Cherniack et al., 2019; Dewa et al., 2019; *n* = 3); and 2018 (McConachie et al., 2018; Rapaport et al., 2018; *n* = 2).

**TABLE 3:** Overview of the main findings from included studies (*n* = 16).

Author (year) *country*	Study design	Co‐production methodology used					Sleep‐specific research		Results/conclusions from the study
		Component	Type	Description	Participants	Study stage	Component	Description	
Alamoudi et al. (2024) *UK*	COP; Qual; Feas	Usability of a mental health app	Focus groups	Focus groups to inform app *Main questions*: How do you usually wake up every day? Do you rely on an alarm? Or do you check your phone? What features do you think are important for young people to engage with the app? Do you think a silent app in the background is best? Or an app that you input into? Do you think young people will accept their data being collected?	*Focus group* (*n* = 7; 18–25 years; 2M/5F) *Feasibility study* (*n* = 11; 18–25 years; 4M/7F)	Study design and feasibility	Component of a mental health app	App includes sleep measures *Sleep disturbance*: An algorithm including three periods of phone usage over 5 min per night, for 5 nights out of 7, or a total of three periods of > 20 phone movements within 30 min per night, for 5 nights out of 7 *Sleep condition indicator*: Assesses insomnia disorder‐based DSM‐5 criteria (scores 0–10, higher scores indicating better sleep)	(1) The app can detect insomnia at an early stage (2) Mobile phone sensors can be used to predict mental health problems that influence sleeping patterns, but further research is needed to optimise its accuracy and validate its effectiveness
Andemeskel et al. (2023) *USA*	COP; Qual	Branding, study design of a healthy lifestyles intervention	Focus groups; interviews; community engagement events	Community engagement strategies to inform and enhance: the study website, recruitment resources, hiring a dedicated engagement coordinator/community health educator as a member of the team, working with the Helen Diller Family Comprehensive Cancer Center Office of Community Engagement and Community Advisory Board members; presenting the educational, research and study recruitment materials at community events; and establishing a community advisory group specifically for the study, 20 semi‐structured user testing interviews were conducted with diverse cancer survivors to inform the look, feel and usability of the study website Interview themes: Content (clarity, informative, relevance), usability (interface) and visuals (layout, colours and imagery)	*Interviews* (*n* = 20; 20–80 years; 14M/6F)	Study design	Component of a healthy lifestyle intervention	Intervention focuses on diet, exercise and sleep Community partnerships and interviews assisted with decisions the study name, Eat Move Sleep Study (EMOVES), logo, redesigning the study website, and study format	(1) Including an engagement coordinator as a permanent team member, partnering with the institutional community outreach and engagement resources (i.e. OCE), and allocating dedicated time and financial support for cultivating relationships with stakeholders outside the university were critical to the development of the study (2) The team successfully engaged with the community to guide pilot work for developing a future cohort study of diverse cancer survivors
Bedson et al. (2019) *UK*	COP; Qual; Feas	Usability of a pain monitoring app	Workshop (researcher group)	*Development of the app*: Research workshops were used to determine content, question phrasing, response options, and appearance and functionality of the app. Consensus on the final content was achieved through the use of electronic voting and ranking options in order of preference *Feasibility, acceptability and clinical utility* (1) Using the app to answer a baseline and follow‐up questionnaire (2) Post‐app follow‐up, a workshop was organised with study participants, which was facilitated by members of the research team, and semi‐structured telephone interviews to discuss acceptability, feasibility and utility	*App development*: (*n* = 9 researchers) *Feasibility, acceptability and clinical utility*: GPs (*n* = 17), nurse practitioners (*n* = 2), patients (*n* = 25; 18+ years with a new episode of musculoskeletal pain)	Study design and feasibility	Component of an app monitoring pain	One of the six questions used to assess pain severity, monitors the impact of pain on sleep Sleep domain: “Level of pain interference or had the pain disturbed sleep” Other domains include sites of pain, wellbeing, medication, side‐effects	(1) Development of the Keele Pain Recorder tablet app, which both patients and clinicians considered easy to use (2) Early testing shows promise in terms of validity, acceptability and clinical usefulness, with clear priorities identified for further testing and investigation
Brown et al. (2024) *UK*	COP; Qual; Feas	Design and testing of a fatigue reducing intervention	Workshops (lived experience patients, carers, clinicians)	*Intervention development*: Three workshops were conducted with people experiencing fatigue after critical illness, family members, and clinicians develop a first intervention draft *Feasibility*: Intervention covers four key topics over a 6‐week period with facilitator support but may continue to be used over a longer period. Two support allowing users to first orient themselves to the intervention and plan goals in week 2 and later “check in” with the facilitator to reflect and discuss progress in week 6. Users will complete a formal fatigue assessment at the beginning and end of the intervention, with shorter prompts after completion of each topic to reflect on how they feel and how they are using the strategies	*Intervention development*: Lived experience participants (*n* = 7), carer/family member (*n* = 1), critical care clinicians (*n* = 7) *Feasibility*: Lived experience participants (*n* = 4; 21–63 years; 1M/3F)	Study design and feasibility	Component of an intervention to reduce fatigue	One of the four intervention topics covered using education‐based resources (website and educational documents) included “strategies for everyday life (covering physical activity; home life; leisure and relationships; work, study and finances; thoughts and feelings; sleep and eating)”, and how sleep and eating can be affected by fatigue, advice for sleep hygiene, strategies for maintaining nutrition when fatigued (via text, videos and infographics) Other topics covered include information on fatigue, managing energy levels and goal setting	(1) Production and testing of the intervention (2) Highlights the benefits of stakeholder involvement to ensure interventions are informed by user needs (3) The intervention shows promise as a self‐management tool for people with fatigue after critical illness. It has the potential to provide education and strategies to patients at the point of discharge and follow‐up
Caswell et al. (2020) *UK*	COP; Qual	Product review, guidance and feedback	Focus groups (children and parents with lived experience)	To discuss previous alarm use, things that were important to them with regard to alarm design/use, and to provide feedback specifically on the new MyPAD alarm system, which was in the early stage of development	Children (and their parents) with nocturnal enuresis (*n* = 3), nurse (*n* = 1), MyPAD design engineers (*n* = 2)	Product development	Outcome of the focus group; development of medical device used during sleep	Sleep disturbance came out as a theme from focus groups of what the product should consider and reduce. Outputs: “all children liked a ‘boxer shorts’ cut, and were hugely insistent that the garment should be comfortable to wear and should not disturb the child's sleep as a consequence of its design”	(1) Importance of PPI in the early design stage of medical devices (2) Evaluation of the MyPAD has prompted the consideration of changes to some existing facets of the device, including providing multiple alarm types, more options for the design of the garment that houses the device, and the need for clear, age‐appropriate and informative instructions
Cherniack et al. (2019) *USA*	COP; Qual	Identify priority concerns for prison staff for future research	Focus groups (employees)	Employee focus groups and surveys: to highlight areas of concern across four topics: overtime and sleep, work–family balance, physical fitness, and mental health; these were later expanded to eight priority areas. Quantitative rankings were generated by focus groups of line‐level employees and supervisors. A multi‐level, iterative priority selection process averaged focus group ratings of topic importance and also difficulty to address separately. Areas of job stress and mental health had highest importance but were also considered most difficult to address	Five focus groups (*n* = 24: COs [*n* = 16] and supervisors [*n* = 8])	Conceptualisation	One of the domains of outcomes consulted in focus group	The components identified as a prioritisation for focus group consultation are overtime and sleep Excessive overtime and reduced sleep quality and quantity appeared to be responsible for some of the worsening health observed in Health Improvement Through Employee Control	(1) Priorities for the following list of eight topics for interventions: fitness and health culture in corrections; overtime and sleep; work–family conflict; mental health of COs in a violent culture; financial stress and job security; managing inmate mental health; effects of violent incidents on workforce; and corrections (2) Balancing participant autonomy and efficient prioritisation of topics among multiple interest groups in this PAR effort met research methods needs and also made it easier for design teams to focus on the difficult and stigmatised area of mental health in the correctional workforce
Crudgington et al. (2020) *UK*	COP; Qual	Identify priority concerns for children and caregivers with epilepsy for future research	Focus groups (children and caregivers with lived experience)	Children and caregivers were consulted to gain an understanding of the acceptability of the two leading outcomes: Quality of Life in Childhood Epilepsy (QOLCE‐55) and Health‐Related Quality of Life Measure for Children with Epilepsy (CHEQOL) Thirty‐eight outcomes were consulted on across 10 domains: (1) Seizures; (2) Sleep; (3) Physical functioning; (4) Social functioning; (5) Behaviour; (6) Mental health; (7) Cognition; (8) Family functioning; (9) Adverse events; and (10) Global quality of life	Children with epilepsy (*n* = 3) and their parents (*n* = 6)	Conceptualisation	One of the domains of outcomes consulted in focus group	Sleep represented one of 10 domains: total time spent asleep at night: total time spent asleep in 24 hr, awakenings from sleep, breathing difficulties, daytime sleepiness, general sleep	(1) Research should consider matching the implicit conceptual framework to the outcomes of research interest and the appropriateness and acceptability of the questionnaire and individual questions to the respondents in the same context that the research will be conducted (2) Researchers should consult with families in the context of PPI/E to ensure measures are acceptable for their settings and their research questions
Dewa et al. (2019) *UK*	COP; Qual	Co‐design and co‐research a qualitative research study around mental health deterioration and sleep	Co‐produced qualitative study; delivery and analysis of semi‐structured interviews by co‐researchers	*Co‐production of the study*: Young person design team co‐created the interview topic guide and co‐designed the information sheet, consent form and protocol. Subsequent changes were made to the documentation upon feedback (e.g. language was put in plain English) *Co‐produced study delivered by the co‐researchers*: Young person design team members became paid co‐researchers (*n* = 3), were trained up, conducted interviews and coded transcripts	Young people with lived experience (*n* = 7; 18–25 years)	Study design, data collection and analysis	Outcome of the qualitative study	Main themes from the semi‐structured interviews included using the wearables to detect sleep and the relationship with subsequent mental health deterioration (1) Change in sleep patterns (e.g. sleeping too much or not sleeping enough) was the most commonly reported sign of mental health deterioration. For example, most participants mentioned that having trouble sleeping was always an indicator that their mental health was getting worse (2) Monitoring sleep and activity levels over time using wearables, mobile app and monitoring online social media activity (e.g. change of: uploaded content, frequency of postings, etc.) was generally deemed feasible and not reliant on the person to self‐report	(1) Wearables and/or mobile apps using regular real‐time feedback to detect worsening mood, sleep and/or activity levels as signs of deterioration, delivered through self‐report or an automated programme (sleep and activity only) were deemed generally acceptable and feasible to young patients, in line with studies involving adult patients (2) Young patients are at risk of significant deterioration in mental state, hospitalisation or self‐harm due to the lack of, or delay in, accessing mental health services. The introduction of technologies could help to fill this gap so that those in need are seen and helped earlier
Garbers et al. (2023), *USA*	COP; Qual; Feas	Co‐design and co‐research a sleep and healthy lifestyles intervention	Advisory groups; focus groups (adolescents, parents)	*Step 3*: Interdisciplinary expert team including adolescent advisors with lived experience aided in the creation of research tools (e.g. focus group guides), development of intervention materials (visual logos, handouts, flyers and curricula activities), and reviewed and provided feedback on handouts and infographic visuals and intervention delivery techniques (e.g. including parents or not, delivering mindful breathing in a group or individual setting, language used) *Step 4*: Focus groups with adolescents to inform the design and methodology of intervention sessions *Step 6*: Intervention development: intervention revised by expert team and Adolescent Advisors *Step 7*: Feasibility study with post‐completion feedback to refine methodology *Other steps*: Step 1 (needs assessment), 2 (review), and 5 (qualitative interviews with staff) – no co‐production methodology	*Step 3*: Adolescent advisors (*n* = 2; 15–16 years, 2F) *Step 4*: Adolescents (*n* = 25; 13–17 years) *Step 7*: Adolescents (*n* = 28; 13–17 years)	Study design and feasibility	Intervention	Sleeping Healthy, Living Healthy, an intervention that integrates sleep hygiene and Mind–Body Integrative Health (MBIH) techniques to improve sleep among adolescents living in urban areas. The intervention is composed of 6 × 40 min weekly group sessions including: (1) Sleep basics and mindful breathing; (2) Good sleep practices and acupressure; (3) Sleep routines and tapping; (4) Sleep environment and body awareness; (5) Changing sleep behaviours, letting go techniques and mindful attention; and (6) wrap‐up and celebration. A 1 × 20 min individual session was also given to cover any missed content from the group sessions	(1) Opportunities to use participatory methods to design interventions that improve health outcomes, including sleep for specific populations should be used (2) An urgent call for sleep interventions for adolescents living in urban areas, which can be completed by incorporating novel elements in intervention design, including an interdisciplinary team including adolescent advisors with lived experience, and rigorous qualitative work with both the participants and those delivering the intervention
McConachie et al. (2018), *UK*	COP; Qual	Identify priority concerns for children and caregivers with ASD for future research	Workshops (parents)	Sixty‐two outcome constructs collected from a systematic review on the requirements of the parents of children with ASD when designing interventions were discussed and prioritised by parent advisory groups	Seventeen parents of children with ASD	Conceptualisation	Outcome of the qualitative study	Sleep was discussed and prioritised highly as a problematic habit behaviour, along with diet and food‐related behaviours and sensory processing issues Sleep problems was identified as an activity level indicator, and suggested as an outcome to be measured alongside school readiness and aggression	The outcome with the highest level of consistency was that sleep problem measures should be a high outcome priority for interventions
Namazi et al. (2023), *USA*	COP; Int; Qual	Co‐design an intervention for improving sleep and health in prison staff	Advisory groups (employees)	*Step 1*: Identify health and safety problems and contributing factors *Step 2*: Set measurable objectives and brainstorm solution activities *Step 3*: Set selection criteria for evaluating solution activities *Step 4*: Apply selection criteria and create three intervention alternatives *Step 5*: Rate interventions	*Step 1* (*n* = 7, meetings = 2) *Step 2* (*n* = 5, meetings = 2) *Step 3* (*n* = 6, meetings = 2) *Step 4* (*n* = 9, meetings = 1) *Step 5* (*n* = 5, meetings = 1)	Study design	Intervention	*Step 1*: Health and safety problem: poor sleep quantity and quality and the contributing factors *Step 2*: Health and safety goal: increase reported sleep hours and ratings of sleep quality. Solution activities include mindfulness and environmental solutions and a decrease in work hours *Step 3*: Criteria required for a favourable evaluation of intervention: impact/scope, benefits/effectiveness, resources/costs, obstacles/barriers *Step 4*: Intervention A: Mind/body Training Session Intervention B: Environmental Training Session Intervention C: Increase Sleep Hours/Decrease Work Hours *Step 5*: Interventions A and B are first priority (equally prioritised) and Intervention C activities are second priority	(1) These are the first sleep improvement interventions designed for correctional middle managers in which the responsibility for intervention development and implementation rests on the managers themselves (2) The interventions selected for implementation are promising in their potential for improving health and safety outcomes due to their grounding in the lived experience of supervisors and concentration on multiple underlying root causes
Perkes et al. (2022), *Australia*	COP; Qual	Consultation on mental health app development	Focus groups (lived experience, clinicians); interviews (lived experience)	Discussions and activities were used to identify: (1) design characteristics; (2) content modules; and (3) features and functions Focus groups and interviews: Mothers discussed three questions (1) How would a mHealth intervention designed for healthy living for Aboriginal and Torres Strait Islander people differ from other mHealth interventions? (2) Are you more interested in mHealth for your own health or your child's health? (3) What topics and features interest you? Follow‐up topics of discussion based on initial responses included: (1) What do you think stops or prevents some women from accessing health information and services for themselves and their children? (2) What do you think are the most important health and well‐being topics to include for Aboriginal or Torres Strait Islander women, children and family? (3) What are the barriers for Aboriginal or Torres Strait Islander families to having good health? (4) What types of mobile technology do you think could support Aboriginal or Torres Strait Islander women's and children's health?	Eight focus groups (*n* = 31 lived experience, 11 clinicians) interviews (*n* = 12 lived experience) interviews	Study design	Component of intervention to improve mental health	Sleep was one of the six modules incorporated for child health within the mHealth intervention	An mHealth intervention that included app, text messaging and Facebook page modalities was developed based on co‐design findings. The intervention incorporates health behaviour change theory, evidence‐based information, and the preferences of Aboriginal and Torres Strait Islander women and health professionals
Rapaport et al. (2018), *UK*	COP; Qual	Consultation of intervention suggests for optimising sleep in participants with dementia	Focus groups (lived experience and caregivers)	Focus groups: Using a semi‐structured guide those with lived experience of dementia to inform and revise future intervention drafts and to share their experiences of strategies they did and did not find useful. Key topics explored: (1) the sleep disturbances people with dementia experienced; (2) the explanations carers had for these difficulties; (3) the effects of these difficulties; (4) what carers found helpful; and (5) DREAMS:START intervention suggestions	*Co‐production team*: Experts in dementia intervention development and care (*n* = 3), manualised cognitive behavioural interventions for sleep disorders (*n* = 2) and Alzheimer's Society research network volunteers with lived experience	Study design and feasibility	Intervention	The DREAMS:START manual was designed to optimise the person with dementia's nighttime sleep and daytime wakefulness. Sessions 1–5 included a range of key topics around sleep knowledge and improving sleep. A specific plan or goal, a stress reduction exercise and sleep diary were devised to be completed between sessions	(1) The DREAMS:START intervention was co‐produced (2) Working with those with “experts by experience” whose lives had been affected by dementia at all stages of the project enabled us to co‐produce a complex and technical intervention in an accessible and usable form (3) Working with “experts by experience” in a co‐production process does not have to be a choice between disregarding the professional academic and clinical expertise or “tokenistic involvement” of PPI partners (3) Ensuring those who participate are given the opportunity to hear how their contribution has made a difference and sharing any outcomes of the research as they become available is a key part of the process
Ryan et al. (2023), *USA*	COP; Qual; Feas	Co‐design and produce resources for internet‐delivered mental health intervention for veterans	Workshops; evaluation panels (veterans, spouses)	Three meetings per panel group *Session 1*: *Veteran*: Perceived motivators, facilitators and barriers to uptake and persistence with self‐help resources on the web *Session 1*: *Spouse*: (1) Their ability and willingness to influence their veterans to seek outside help or resources (particularly web‐based resources); and (2) The process of finding resources and determining which ones they would recommend to their veterans *Session 2*: *Veteran and spouse* The layout, text and functionality of drafts of PTBS content, especially the sleep diary, sleep prescription calculator, and relapse prevention *Session 3: Veteran and spouse*: The layout, text and functionality of drafts of PTBS content, especially the landing page, course guide map, initial learning module, and fact sheet	Veteran panel: active duty experience veterans (*n* = 27, across three groups; 18–70 years; at least 1F per group) Spouse panel: (*n* = 18, across 2 groups; 18–70 years; at least 1 M per group)	Study design and feasibility	Intervention	Internet‐delivered digital mental health intervention adaptation of CBT‐I known as PTBS	(1) Consistent feedback on recommendations to emphasise the efficacy of CBT‐I techniques, improve the design of the intervention and ensure that content is consistent with the lived experiences of veterans was derived (2) Evaluation panels could prove useful to other digital mental health intervention designers
Vandendriessche et al. (2023), *Belgium and The Netherlands*	COP; Qual	Co‐design and co‐research of a school‐based sleep intervention	Workshops; advisory groups (adolescents)	Three intervention schools: in each school, an action team (adolescent advisory group) was composed to develop and plan the intervention using intervention mapping (23–34 weekly workshops). Parental intervention components were developed using a parent PAR group	Adolescent advisory group (*n* = 12 per group; 15 years) Parent advisory team (*n* = 7 per group)	Study design, data collection	Intervention	**Sleep co‐design sessions** Sessions 1–4 (intervention mapping step 1): Intervention team and health problem, discussing photo voice: influencing factors of sleep, setting up small studies to further explore influencing factors, reporting on the small studies, creating list of problems regarding sleep Session 5–6 and 8–10 (intervention mapping step 2): Formulating performance objectives, capacity building: explaining behavioural determinants to the action team, formulating change objectives and selecting methods for each performance objective Session 7–10 (intervention mapping step 3): Introducing methods of behaviour change, formulating change objectives and selecting methods for each performance objective Session 11–14 and 16, 18–19, 21–34 (intervention mapping step 4): specifying practical applications and arranging them on timeline, developing intervention components Session 17 and 20 (intervention mapping step 5): Identifying implementers *Sleep intervention* (1) school posters, (2) class discussions (schedule for class discussions [which topic/which week], background information on sleep and tips for motivational interviewing), (3) sleep app (introducing the app and handing out stickers with a QR code to download the app, reminding to use the app and short explanation of the functions, going from class to help students install the app), (4) class about sleep, (5) class about the link between physical activity and sleep in PE, (6) yoga class to teach relaxation exercises, (7) incentives for those using the sleep app, (8) fitbit class competition (class score created based on criteria set by the action team)	(1) Combining PAR and intervention mapping resulted in interventions focusing on the importance of healthy sleep, regular sleep patterns and associated behaviours: screen behaviours, physical activity, dietary behaviour and relaxation (2) Combining PAR with intervention mapping resulted in more extensive interventions than other existing school‐based sleep interventions
Vera San Juan et al. (2022), *UK*	COP; Qual	Identify priority concerns for children and young people for future research	Focus groups (children, young people)	A three‐stage consensus‐based consultation process Stage 1: Preliminary Consultation to Develop a Long List of Relevant Research Questions: Nine researchers Stage 2: Participatory Discussion Groups to Build on and Refine the Long List: Young people, parents and teachers Stage 3: Public Consultation for Final Prioritisation: 357 completed the main ranking task (229 young people; 128 adults) and 330 (210 young people; 120 adults) answered the open‐ended questions	Children and young people (*n* = 229) and adults (*n* = 128)	Conceptualisation	Priority topic for future research	Sleep was one of 26 research questions of importance that were ranked by participants Sleep and its relationship with mental health was highlighted as a prioritisation for future research	(1) Consensus was reached for the prioritisation of four topics for future research: (i) the impact of exposure to adult content on young people's mental health and relationships; (ii) the relationship between screen use and the well‐being of young people from vulnerable groups; (iii) the impact of screen use on brain development; and (iv) the relationship between screen use and sleep (2) The collaboration with young people and focus on young peoples' views allowed us to identify research priorities on screen use and adolescent mental health, and to gain insight into the reasoning behind these research questions

Abbreviations: ASD, autism spectrum disorder; CBT‐I, cognitive behaviour therapy for insomnia; COP, co‐production; COs, correctional officers; DSM‐5, Diagnostic and Statistical Manual of Mental Disorders, 5th Edition; F, female; Feas, feasibility; Int, intervention; M, male; OCE, office of community engagment; PAR, participatory action research; PE, physical education; PPI, Personal and Public Involvement; PPI/E, Patient and Public Involvement/Engagement; PTBS, path to better sleep; Qual, qualitative.

### 3.3 Data synthesis

The included studies were synthesised by two main components: the co‐production methodologies used; and the component of research that incorporated sleep as an outcome. All 16 studies used a range of co‐production methods, including focus groups (Alamoudi et al., 2024; Andemeskel et al., 2023; Caswell et al., 2020; Cherniack et al., 2019; Crudgington et al., 2020; Garbers et al., 2023; Perkes et al., 2022; Rapaport et al., 2018; Vera San Juan et al., 2022; *n* = 9), workshops (Bedson et al., 2019; Brown et al., 2024; McConachie et al., 2018; Ryan et al., 2023; Vandendriessche et al., 2023; *n* = 5), advisory groups (Garbers et al., 2023; Namazi et al., 2023; Vandendriessche et al., 2023; *n* = 3), interviews (Andemeskel et al., 2023; Dewa et al., 2019; Perkes et al., 2022; *n* = 3), evaluation panels (Ryan et al., 2023; *n* = 1) and community engagement events (Andemeskel et al., 2023; *n* = 1; Table 3). Ten studies used solely methods of qualitative PPI or co‐production methods to inform intervention design and development (Andemeskel et al., 2023; Perkes et al., 2022; Rapaport et al., 2018; Vandendriessche et al., 2023; *n* = 4) or to prioritise research outcomes (Caswell et al., 2020; Cherniack et al., 2019; Crudgington et al., 2020; Dewa et al., 2019; McConachie et al., 2018; Vera San Juan et al., 2022; *n* = 6; Table 3). Six studies used qualitative PPI or co‐production methods and intervention feasibility testing to inform app (Alamoudi et al., 2024; Bedson et al., 2019; *n* = 2) or intervention (Brown et al., 2024; Garbers et al., 2023; Namazi et al., 2023; Ryan et al., 2023; *n* = 4) design, development, feasibility, acceptability and utility (Table 3). Most studies used consultation approaches to design interventions (*n* = 8), instead of co‐design teams (Rapaport et al., 2018; Vandendriessche et al., 2023; *n* = 2).

All 16 studies highlighted how the co‐production methodologies used were useful in informing the design, development and feasibility of interventions methodologies, or in informing research outcomes and recruitment strategies, by amplifying the voice of those affected (Alamoudi et al., 2024; Andemeskel et al., 2023; Bedson et al., 2019; Brown et al., 2024; Caswell et al., 2020; Cherniack et al., 2019; Crudgington et al., 2020; Dewa et al., 2019; Garbers et al., 2023; McConachie et al., 2018; Namazi et al., 2023; Perkes et al., 2022; Rapaport et al., 2018; Ryan et al., 2023; Vandendriessche et al., 2023; Vera San Juan et al., 2022; Table 3). No studies reported co‐production methodologies to be unhelpful or problematic to the research being conducted. The sleep focus of the co‐production research varied across studies, including sleep being a component of an intervention (Alamoudi et al., 2024; Andemeskel et al., 2023; Bedson et al., 2019; Brown et al., 2024; Perkes et al., 2022; *n* = 5), sleep being the main focus of the intervention (Garbers et al., 2023; Namazi et al., 2023; Rapaport et al., 2018; Ryan et al., 2023; Vandendriessche et al., 2023; *n* = 5), and sleep being an outcome of priority for further research (Caswell et al., 2020; Cherniack et al., 2019; Crudgington et al., 2020; Dewa et al., 2019; McConachie et al., 2018; Vera San Juan et al., 2022; *n* = 6; Table 3).

### 3.4 Major themes within current literature

#### 3.4.1 Co‐production methodologies in interventions focusing on sleep

Five studies focused primarily on improving sleep (Garbers et al., 2023; Namazi et al., 2023; Rapaport et al., 2018; Ryan et al., 2023; Vandendriessche et al., 2023). Garbers et al. used adolescent advisory panels and focus groups to inform resource and intervention development for the “Sleeping healthy, living healthy” intervention, followed by a feasibility study within a healthy adolescent population (Garbers et al., 2023). Similarly, Vandendriessche et al. described the intervention mapping of three school interventions using adolescent advisory panels and a co‐design team to inform the development and logistical delivery of a school‐based sleep improvement intervention (Vandendriessche et al., 2023). Unlike Garbers et al. (2023), Vandendriessche et al. (2023) used a co‐design team, with adolescents undertaking a range of team roles to design the interventions as opposed to only consultations and feedback.

Another study aimed to develop a sleep intervention for prison staff, spanning various employee and management roles, utilising multiple advisory group sessions (Namazi et al., 2023). These sessions were used to identify the root causes of poor sleep and fatigue, as well as to design, test and review several intervention options to improve the sleep of staff (Namazi et al., 2023).

The remaining two studies designed sleep‐focused interventions for clinical populations; a cognitive behavioural therapy for insomnia (CBT‐I) intervention for veterans with poor sleep (Ryan et al., 2023) and a sleep intervention (“DREAMS: START”) for patients with dementia (Rapaport et al., 2018). Ryan et al. used multiple workshops to consult veterans and their spouses to develop and test the feasibility of a CBT‐I intervention (Ryan et al., 2023). Similar to the study by Vandendriessche et al. (2023), the DREAMS: START intervention for participants with dementia was co‐designed using a team, as opposed to consultations only, and the feasibility of the intervention was not assessed (Rapaport et al., 2018). The team consisted of a range of clinical experts and Alzheimer's volunteers with lived experience of caring for patients with dementia (Rapaport et al., 2018).

#### 3.4.2 Co‐production methodologies in interventions with a sleep component

The five included studies that researched sleep as a component of an intervention included interventions for mental health (one app‐based intervention [Alamoudi et al., 2024] and one in‐person intervention [Perkes et al., 2022]), a healthy lifestyle in‐person intervention (Andemeskel et al., 2023), musculoskeletal pain app‐based intervention (Bedson et al., 2019), and critical illness fatigue website and resource intervention (Brown et al., 2024).

Both app interventions used focus groups to aid in the design and usability of a health tracking app for detection of poor mental health (Alamoudi et al., 2024), and a pain monitoring and reporting app. Alamoudi et al. included young people in both the focus groups and feasibility testing of a mental health app, which determined the app could detect early insomnia prior to a poor mental health episode (Alamoudi et al., 2024). Bedson et al. used researchers only in the development of the pain monitoring app, and then utilised patients with lived experience of musculoskeletal pain to test the feasibility and acceptability of the app (Bedson et al., 2019). One of the domains used to monitor pain assessed how much the pain disturbed the patient's sleep (Bedson et al., 2019).

Andemeskel et al. used focus groups and interviews with those with lived experience and community engagement events to develop the methodologies used and design of the “Eat Sleep Move Study (EMOVES)” to encourage healthy lifestyles in cancer survivors (Andemeskel et al., 2023). Another intervention designed for a clinical population involved the development of a website and resources to improve self‐management of fatigue in those with critical illness after discharge, including a sleep hygiene component (Brown et al., 2024). The resources and website were co‐designed by a team including those with lived experience, clinicians and carers, and then the feasibility was tested by those with lived experience (Brown et al., 2024). Similarly, a mental health intervention (“mHealth”) was designed using focus groups of Aboriginal and Torres Strait Islander women and clinicians and interviews with the women, to design and develop a mental health intervention for Aboriginal and Torres Strait Islander women and children (Perkes et al., 2022). Sleep hygiene and improvement were one of the six domains for child health. No feasibility assessment was conducted (Perkes et al., 2022).

#### 3.4.3 Co‐production methodologies in studies assessing sleep as a priority outcome for future research

Six studies had sleep as a priority outcome for future intervention research (Cherniack et al., 2019; Crudgington et al., 2020; Dewa et al., 2019; McConachie et al., 2018; Vera San Juan et al., 2022; *n* = 5) and an outcome for product design (Caswell et al., 2020; *n* = 1).

Three studies assessed sleep as a priority outcome for future interventions in clinical populations including children with epilepsy (Crudgington et al., 2020), young people with poor mental health (Dewa et al., 2019), and children with autism spectrum disorder (ASD; McConachie et al., 2018). Crudgington et al. consulted children with lived experience of epilepsy and their parents on 38 different outcomes from two quality of life scales for children with epilepsy (Crudgington et al., 2020). Total sleep time in 24 hr, number of awakenings, breathing difficulties, daytime sleepiness and general poor sleep were key themes from the focus groups as priorities for interventions to improve the quality of life of children with epilepsy (Crudgington et al., 2020). Dewa et al. used a co‐design team of young people with lived experience of poor mental health to co‐produce the methodology and the study outcomes for future intervention research in young people (Dewa et al., 2019). A change in sleep pattens was the most commonly reported sign of poor mental health, and using wearables may be the most acceptable way to capture sleep patterns in this population (Dewa et al., 2019). Another study used focus groups with the parents of children with ASD to review 62 outcome constructs for future research in children with ASD that had been highlighted as future focus outcomes in systematic reviews (McConachie et al., 2018). The outcome with the highest level of consistency to be focused on in future interventions was sleep problems (McConachie et al., 2018).

Two other studies assessed the prioritisation of research outcomes in health populations, one in prison staff (Cherniack et al., 2019) and another in young people (Vera San Juan et al., 2022). Cherniack et al. reported on areas of concern for employees at prisons, with overtime and poor sleep quality and quantity being a main theme identified as responsible for worsening health in staff and for future interventions (Cherniack et al., 2019). Vera San Juan et al. used a three‐stage consensus to rank outcomes of priority for future research in young people (Vera San Juan et al., 2022). Outcomes were first determined by an expert panel of researchers, built upon by a focus group of young people, parents and teachers, and then prioritised by the public (children and adults) who ranked outcomes by importance (Vera San Juan et al., 2022). The relationship between screentime use and sleep was highlighted as an area of public interest for further research (Vera San Juan et al., 2022).

Caswell et al. conducted focus groups of children with lived experience of nocturnal enuresis, their parents, a specialist nurse and research engineers to review the “MyPAD” alarm pad for nocturnal enuresis (Caswell et al., 2020). When reviewing the product, sleep disturbance came out as the top priority for the product to reduce, and that the product should be comfortable and not disrupt sleep across the night (Caswell et al., 2020).

## 4 DISCUSSION

### 4.1 Overview of findings

This is the first scoping review that aims to identify and synthesise the use of co‐production methodology within sleep research (we did not identify any studies incorporating co‐production in circadian research). Broadly, the studies used multiple different components of co‐production methodologies; however, no studies used co‐production from conceptualisation of the research (beginning) to the dissemination of the research (end). Many of the studies had sleep as a secondary outcome, suggesting that the primary focus of the research was within a different field, for example, mental health (Alamoudi et al., 2024; Andemeskel et al., 2023; Bedson et al., 2019; Brown et al., 2024; Perkes et al., 2022). Consequently, this could be misleading on co‐production use in sleep‐specific research as co‐production practices may be more common in those fields. This highlights the limited published evidence of using co‐production methodologies in sleep research and the insufficient use of standardised frameworks.

Sharing best practices from fields like mental health may help establish effective co‐production methods and improve the quality and impact of sleep research. Mental health research has advanced significantly in integrating co‐production methodologies, involving individuals with lived experience at every stage of the research process, from conceptualisation to dissemination (Jakobsson et al., 2023). A co‐production in mental health research framework has recently been proposed, drawing on both the successes and challenges encountered in mental health research (Capobianco et al., 2023). This approach has been applied in the development of interventions, digital apps and policy changes, ensuring that outcomes are relevant and effective. In contrast, sleep research has lagged in integrating co‐production, often limiting participation to consultation stages rather than holistic stakeholder involvement. To enhance the relevance and impact of sleep research (especially when sleep interventions are the focus), sleep research could follow the example set by the field of mental health and embed co‐production principles at every stage.

### 4.2 Co‐production methodologies informing sleep research using key stakeholders

All included studies conducted elements of co‐production with key stakeholders; specifically, 10 studies focused on co‐producing apps or interventions that included sleep outcomes (Alamoudi et al., 2024; Andemeskel et al., 2023; Bedson et al., 2019; Brown et al., 2024; Garbers et al., 2023; Namazi et al., 2023; Perkes et al., 2022; Rapaport et al., 2018; Ryan et al., 2023; Vandendriessche et al., 2023), either as a primary or secondary focus (Alamoudi et al., 2024; Andemeskel et al., 2023; Bedson et al., 2019; Brown et al., 2024; Garbers et al., 2023; Namazi et al., 2023; Perkes et al., 2022; Rapaport et al., 2018; Ryan et al., 2023; Vandendriessche et al., 2023), and six studies identified priority outcomes for sleep research (Caswell et al., 2020; Cherniack et al., 2019; Crudgington et al., 2020; Dewa et al., 2019; McConachie et al., 2018; Vera San Juan et al., 2022). Key stakeholders that were consulted or used in co‐production in the included studies were: researchers, policy makers, clinical staff, teachers, prison staff, children, young people and caregivers. For example, Cherniack et al. involved prison staff in identifying poor sleep as a key health concern for future interventions (Cherniack et al., 2019), while Crudgington et al. consulted children with epilepsy and their parents to highlight sleep‐related challenges such as awakenings and daytime sleepiness (Crudgington et al., 2020). Dewa et al. engaged young people with lived experience of poor mental health to co‐produce sleep‐related outcomes, suggesting that wearables could effectively monitor sleep patterns in future interventions (Dewa et al., 2019). These studies demonstrate how engaging stakeholders—whether they are participants, caregivers, or those who work closely with the participant demographic—can shape the research process to better reflect real‐world needs with applicable outcomes (Vargas et al., 2022).

Lived experiences related to sleep research was defined as those with sleep disturbances, sleep disorders like insomnia, mental health disorders or other health measures, as well as those with good sleep. Engaging these groups ensures that the perspectives of those directly involved with or working alongside the participant demographic under investigation are fully considered and thus leading to more relevant and effective interventions. Co‐production principles that meaningful stakeholder involvement must span the entire research process, from conceptualisation to dissemination, in order to have a significant impact (Smith et al., 2023). Yet, most of the included studies used focus groups or advisory panels (Alamoudi et al., 2024; Andemeskel et al., 2023; Brown et al., 2024; Caswell et al., 2020; Cherniack et al., 2019; Perkes et al., 2022; Rapaport et al., 2018; Ryan et al., 2023; Vandendriessche et al., 2023; Vera San Juan et al., 2022), which often restricted stakeholders to providing feedback rather than actively participating in decision‐making (Galende‐Sánchez & Sorman, 2021). For co‐production to be truly effective, stakeholders should be involved in every stage, including the design, data collection, analysis and dissemination phases (Smith et al., 2023). Expanding their role beyond consultation to active collaboration ensures that their lived experiences and insights shape the research in a more integrated and impactful way, driving relevance and innovation in both methodology and outcomes. By allowing the research team to retain control over key decisions, many of these studies fall short of the core principle of co‐production, which seeks to foster shared ownership of the research process (Smith et al., 2023). This gap is further highlighted by the lack of focus on dissemination strategies or discussions about the impact of the interventions and apps being developed (Harcourt & Crepaz‐Keay, 2023). Expanding co‐production beyond consultation into genuine collaboration would enhance the quality, relevance and long‐term impact of sleep research interventions (Galende‐Sánchez & Sorman, 2021).

### 4.3 Co‐producing sleep research with children and young people

Co‐producing sleep research with children and young people was a key focus in seven of the included studies (Alamoudi et al., 2024; Caswell et al., 2020; Crudgington et al., 2020; Dewa et al., 2019; Garbers et al., 2023; Vandendriessche et al., 2023; Vera San Juan et al., 2022), with an additional four studies that consulted parents about their child's sleep (Caswell et al., 2020; Crudgington et al., 2020; McConachie et al., 2018; Vandendriessche et al., 2023). Notably, the only studies that incorporated co‐researchers—where children actively contributed to the development and implementation of the intervention or research study—were conducted in school settings (Garbers et al., 2023; Vandendriessche et al., 2023) or recruited via charities (Dewa et al., 2019). These studies, such as those by Garbers et al. (2023) and Vandendriessche et al. (2023), involved adolescents taking on active roles in intervention design, showcasing a high level of youth participation and true co‐production.

The significance of co‐production with adolescents extends beyond sleep research, aligning with broader frameworks like the UN Convention on the Rights of the Child (Pais & Bissell, 2006), which encourages hearing the voices of young people in all areas of research. Hart's (1992) Ladder of Children's Participation supports this approach by advocating for higher levels of involvement, where children initiate decisions and share control with adults (Hart, 1992). The school‐based interventions that utilised co‐researchers are examples of this higher level of participation, as they directly included young people in shaping the study. Applying these practices in adult sleep and circadian research could provide a pathway for more inclusive and effective co‐production, ensuring that participants are engaged as co‐creators in the research process.

Incorporating young people into the research process, as demonstrated in school settings, highlights how co‐production can enhance the design and relevance of sleep interventions. This approach is especially valuable in sleep research, where invasive and longitudinal data collection methods (Ibáñez et al., 2018) may present challenges for engagement and result in high attrition, especially with adolescents (Lyall et al., 2020). By involving young people in decision‐making about the methods, duration and procedures for collecting sleep data, researchers can create more feasible and participant‐friendly studies (Tisdall, 2017), particularly when the needs of adolescents differ from those of adults. Empowering young participants as co‐researchers enhances not only retention and engagement but also the real‐world applicability and success of sleep interventions (Murray & Xie, 2024).

### 4.4 Strengths and limitations of co‐production studies identified in sleep research

Co‐production methodologies offer unique benefits, particularly in sleep research, where the collaborative nature of co‐production allows for the incorporation of diverse perspectives and experiences. These methodologies emphasise stakeholder involvement, including individuals with lived experience, which ensures that research outputs are more relevant and grounded in real‐world experiences. This approach fosters greater alignment between the research aims and the needs of the community, leading to the development of more tailored and effective interventions. Importantly, co‐production allows for flexibility, enabling the research to adapt to evolving circumstances, such as the challenges posed by the COVID‐19 pandemic. In addition, the iterative nature of co‐production ensures that the findings reflect the collective input of various stakeholders, rather than solely the perspectives of researchers. Despite often employing smaller sample sizes, co‐production aims for saturation—the point at which no new themes emerge—ensuring a comprehensive understanding of the issues being studied (Malterud et al., 2016).

However, several theoretical and methodological limitations were highlighted in the studies. Specifically, the limitations identified were small sample sizes (*n* = 14), researcher‐driven topics or domains in the co‐production process (*n* = 6), lack of a representative sample (*n* = 6), and challenges in conducting face‐to‐face sessions due to COVID‐19 (*n* = 4). Some studies called for larger (*n* = 9) or more diverse (*n* = 6) samples, or broader inclusion across different communities (*n* = 4), to ensure that the results fully represent the population and capture the wide range of lived experiences.

In addition to these practical limitations, there was also a recurring issue with the lack of clarity surrounding what co‐production entails and how it should be implemented during various research stages. This uncertainty extends to distinguishing co‐production from related methodologies, such as PAR, PPI and PPI/E. Many studies utilised advisory boards or focus groups, but other co‐production strategies, such as involving stakeholders as co‐designers and co‐researchers, were under‐utilised. In several cases, final decisions rested with the researchers rather than the co‐producers, indicating a partial rather than full integration of co‐production principles. Furthermore, many studies lacked a feedback loop to ensure that the research outcomes genuinely reflected the co‐producers' lived experiences. The absence of a clear definition of “lived experience” further limited the efficacy of these approaches, underscoring the need for clearer guidelines for consistent application of co‐production methodologies (Ärleskog et al., 2021).

### 4.5 Strengths and limitations of the scoping review

This scoping review was the first to investigate the use of co‐production methodologies in sleep research. Over the last 5 years there has been an increased demand for co‐production methodologies to be incorporated in basic and clinical science, which has been successfully adopted in some lines of research, such as mental health research (Norton, 2021). This review underscores the necessity for sleep research to advance and align with areas like mental health research, in order to enhance the quality, relevance and prioritisation of sleep studies. Another strength of this review was the breadth of study designs highlighting the different methodologies by which co‐production is being used in sleep research. Whilst identifying standard approaches to use in sleep research would be useful, this review highlights that co‐production can be used at multiple stages of research design and implementation.

A limitation of the screening process was that many qualitative studies used terms such as feedback, focus groups and advisory panels to describe the sharing of lived experiences, without indicating meaningful contribution to the co‐production of outcomes, research or products. This reflects a broader challenge in the field, where the inconsistent use of terminology and lack of clear assessment of co‐production impact makes it difficult to identify true co‐production efforts and gauge their effectiveness. Additionally, Delphi studies were excluded from this review because they primarily focused on consultation and feedback of “experts by knowledge” rather than co‐production with “experts by experience”. However, there are blurred lines in research regarding whether Delphi studies, when involving fellow researchers, could be considered a form of co‐production. Consequently, future research should consider establishing a clear definition of co‐production and exploring how best to implement it effectively. Given that co‐production is an elaborate and resource‐demanding addition to the research cycle, a consensus or stepped approach may be necessary to guide its application. Where possible, researchers must account for the additional resources, time and effort required to incorporate high‐quality co‐production processes into their studies. It is also highly probable that co‐production studies are subject to publication bias and, thus, a high majority is not published or shared within the field of sleep research and subsequently not included in this scoping review due to “file drawer bias”.

### 4.6 Recommendations for the use of co‐production methodologies in sleep research

Co‐production as a methodology is gaining traction due to funding bodies increasing the inclusion of lived experiences in research as a requirement. A scoping review by Smith et al. (2022) identified lessons on how co‐production should be embedded within research: (1) the capacity to implement co‐produced interventions; (2) the skill set needed for co‐production; (3) multiple levels of engagement and negotiation; and (4) funding and institutional arrangements for meaningful co‐production. There were identified challenges of implementing co‐production into research such as the time taken, cost and organisational support. However, future research should address such concerns by adapting generic frameworks on who to involve in co‐production, how best to implement co‐production methodologies, the language used to describe co‐production methodologies, and the best practice components of co‐production. Additionally, assessing the impact of co‐production is considered good practice, and can range from protocol modifications to influencing policy changes. In health and social care research, the GRIPP2 tool is recommended to enhance transparency and reporting of PPI (Staniszewska et al., 2017). Both the short and long versions of this checklist outline key elements that should be reported when discussing co‐production. It is advisable to incorporate impact assessment tools, such as GRIPP2, when co‐production is integrated into the research design to ensure comprehensive evaluation and reporting.

The primary recommendation from this review is the need to adapt existing generic frameworks that clearly define co‐production methodologies, providing examples of when and how co‐production can be integrated into various stages of sleep research and extended to studies investigating circadian parameters. This framework should consider the sample population of interest, such as clinical or general groups, the age of the co‐producers involved, like adolescents, and the inclusion of multiple stakeholders, including parents, children, school staff, general practitioners, carers, researchers, product designers and others. Moreover, a recent article highlighted that while generic best practice guidelines offer useful principles, they are often too high level to be fully applicable to sleep‐specific practices, suggesting that tailored frameworks for sleep research may also need to be developed as a result (Power et al., 2024).

### 4.7 Conclusion

There is a growing emphasis on incorporating co‐production methodologies into sleep research, drawing attention to the need for more structured and meaningful stakeholder involvement. While the studies included in this review demonstrated efforts to engage key stakeholders—such as researchers, clinicians, policymakers, caregivers and participants—many relied on consultation rather than full co‐production. The findings underline the importance of moving beyond advisory roles and integrating stakeholders as co‐researchers throughout the research process, from design to dissemination.

The review also illustrates that co‐production in sleep research is still in its early stages, often borrowing methods from fields like mental health and software development where co‐production has been more thoroughly developed. However, the lack of consistent frameworks, guidelines and clarity around the use of co‐production in sleep research remains a significant limitation. Future research must prioritise the adaptation of existing generic frameworks that define co‐production practices, establish clear roles for stakeholders, and standardise methodologies to enhance the relevance, effectiveness and real‐world impact of sleep interventions. By embedding genuine co‐production practices, sleep research can achieve more meaningful, participant‐centred outcomes that better reflect the needs of the populations being studied.

## AUTHOR CONTRIBUTIONS

**Emma Louise Gale:** Conceptualization; investigation; methodology; data curation; writing – review and editing; visualization; writing – original draft; formal analysis. **Raahat Manrai:** Conceptualization; methodology; writing – original draft; writing – review and editing. **Lorna Caddick:** Conceptualization; methodology; writing – original draft; writing – review and editing. **Aja Murray:** Conceptualization; supervision; writing – review and editing; funding acquisition. **Heather C. Whalley:** Funding acquisition; conceptualization; writing – review and editing; supervision. **Daniel Smith:** Conceptualization; funding acquisition; writing – review and editing; supervision. **Maria Gardani:** Conceptualization; writing – original draft; writing – review and editing; funding acquisition; supervision; methodology.

## FUNDING INFORMATION

Wellcome (AMBIENT‐BD project: 226944/Z/23/Z), Medical Research Council (AMBIENT‐Teens project: MR/X028917/1).

## Supporting information


**DATA S1.** Checklist.

## Data Availability

Data sharing not applicable to this article as no datasets were generated or analysed during the current study.

## References

[jsr14476-bib-0001] Alamoudi, D. , Nabney, I. , & Crawley, E. (2024). Evaluating the effectiveness of the SleepTracker App for detecting anxiety‐ and depression‐related sleep disturbances. Sensors (Basel), 24(3), 722. 38339439 10.3390/s24030722 PMC10856976

[jsr14476-bib-0002] Albert, A. , Islam, S. , Haklay, M. , & McEachan, R. R. C. (2023). Nothing about us without us: A co‐production strategy for communities, researchers and stakeholders to identify ways of improving health and reducing inequalities. Health Expectations, 26(2), 836–846. 36683204 10.1111/hex.13709 PMC10010091

[jsr14476-bib-0003] Andemeskel, G. , Palmer, N. R. , Pasick, R. , van Blarigan, E. L. , Kenfield, S. A. , Graff, R. E. , Shaw, M. , Yu, W. , Sanchez, M. , Hernandez, R. , Washington, S. L., III , Shariff‐Marco, S. , Rhoads, K. F. , & Chan, J. M. (2023). Engaging communities to inform the development of a diverse cohort of cancer survivors: Formative research for the eat move sleep study (EMOVES). Research Involvement and Engagement, 9(1), 117. 38082391 10.1186/s40900-023-00529-z PMC10712178

[jsr14476-bib-0004] Ärleskog, C. , Vackerberg, N. , & Andersson, A.‐C. (2021). Balancing power in co‐production: Introducing a reflection model. Humanities and Social Sciences Communications, 8(1), 1–7.38617731

[jsr14476-bib-0005] Arnardottir, E. S. , Islind, A. S. , & Óskarsdóttir, M. (2021). The future of sleep measurements: A review and perspective. Sleep Medicine Clinics, 16(3), 447–464. 34325822 10.1016/j.jsmc.2021.05.004

[jsr14476-bib-0006] Bedson, J. , Hill, J. , White, D. , Chen, Y. , Wathall, S. , Dent, S. , Cooke, K. , & van der Windt, D. (2019). Development and validation of a pain monitoring app for patients with musculoskeletal conditions (the Keele pain recorder feasibility study). BMC Medical Informatics and Decision Making, 19(1), 24. 30683106 10.1186/s12911-019-0741-z PMC6347830

[jsr14476-bib-0007] Beresford, P. , & Beresford, P. (2021). COVID‐19 and co‐production in health and social care research, policy, and practice: Volume 1: The challenges and necessity of co‐production. Policy Press.

[jsr14476-bib-0008] Brown, S. E. , Shah, A. , Czuber‐Dochan, W. , Bench, S. , & Stayt, L. (2024). Fatigue after CriTical illness (FACT): Co‐production of a self‐management intervention to support people with fatigue after critical illness. In Intensive & critical care nursing (Vol. 82, p. 103659). Elsevier. 38401405 10.1016/j.iccn.2024.103659

[jsr14476-bib-0009] Calhoun, S. L. , Pearl, A. M. , Fernandez‐Mendoza, J. , Durica, K. C. , Mayes, S. D. , & Murray, M. J. (2020). Sleep disturbances increase the impact of working memory deficits on learning problems in adolescents with high‐functioning autism spectrum disorder. Journal of Autism and Developmental Disorders, 50(5), 1701–1713. 30788649 10.1007/s10803-019-03928-y

[jsr14476-bib-0010] Capobianco, L. , Faija, C. , Cooper, B. , Brown, L. , McPhillips, R. , Shields, G. , & Wells, A. (2023). A framework for implementing patient and public involvement in mental health research: The PATHWAY research programme benchmarked against NIHR standards. Health Expectations, 26(2), 640–650. 36625226 10.1111/hex.13676 PMC10010097

[jsr14476-bib-0011] Caswell, N. , Kuru, K. , Ansell, D. , Jones, M. J. , Watkinson, B. J. , Leather, P. , Lancaster, A. , Sugden, P. , Briggs, E. , Davies, C. , Oh, C. , Bennett, K. , & DeGoede, C. (2020). Patient engagement in medical device design: Refining the essential attributes of a wearable, pre‐void, ultrasound alarm for nocturnal enuresis. Pharmaceutical Medicine, 34(1), 39–48. 31970684 10.1007/s40290-019-00324-w

[jsr14476-bib-0012] Causer, H. , Spiers, J. , & Riley, R. (2024). A method for synthesizing qualitative data sources in the co‐production of postvention guidelines for the NHS: A worked example. International Journal of Qualitative Methods, 23, 16094069241229985.

[jsr14476-bib-0013] Chen, Y. , Zhou, E. , Wang, Y. , Wu, Y. , Xu, G. , & Chen, L. (2023). The past, present, and future of sleep quality assessment and monitoring. Brain Research, 1810, 148333. 36931581 10.1016/j.brainres.2023.148333

[jsr14476-bib-0014] Cherniack, M. , Berger, S. , Namazi, S. , Henning, R. , Punnett, L. , & CPH‐NEW Research Team . (2019). A participatory action research approach to mental health interventions among corrections officers: Standardizing priorities and maintaining design autonomy. Occupational Health Science, 3(4), 387–407. 37180051 10.1007/s41542-019-00051-3 PMC10174268

[jsr14476-bib-0015] Coldham, T. (2021). National Institute for Health Care Research INVOLVE Advisory Board, *Guidance on co‐producing a research project (INVOLVE 2020)* . National Institute for Health Care Research.

[jsr14476-bib-0016] Cowdell, F. , Dyson, J. , Sykes, M. , Dam, R. , & Pendleton, R. (2022). How and how well have older people been engaged in healthcare intervention design, development or delivery using co‐methodologies: A scoping review with narrative summary. Health & Social Care in the Community, 30(2), 776–798. 33103313 10.1111/hsc.13199

[jsr14476-bib-0017] Crudgington, H. , Collingwood, A. , Bray, L. , Lyle, S. , Martin, R. , Gringras, P. , Pal, D. K. , & Morris, C. (2020). Mapping epilepsy‐specific patient‐reported outcome measures for children to a proposed core outcome set for childhood epilepsy. Epilepsy & Behavior, 112, 107372. 32906016 10.1016/j.yebeh.2020.107372 PMC7689576

[jsr14476-bib-0018] de Gans, C. J. , Burger, P. , van den Ende, E. S. , Hermanides, J. , Nanayakkara, P. W. B. , Gemke, R. J. B. J. , Rutters, F. , & Stenvers, D. J. (2024). Sleep assessment using EEG‐based wearables ‐ a systematic review. Sleep Medicine Reviews, 76, 101951. 38754209 10.1016/j.smrv.2024.101951

[jsr14476-bib-0019] Dewa, L. H. , Lavelle, M. , Pickles, K. , Kalorkoti, C. , Jaques, J. , Pappa, S. , & Aylin, P. (2019). Young adults' perceptions of using wearables, social media and other technologies to detect worsening mental health: A qualitative study. PLoS One, 14(9), e0222655. 31532786 10.1371/journal.pone.0222655 PMC6750581

[jsr14476-bib-0020] Dregan, A. , & Armstrong, D. (2011). Cross‐country variation in sleep disturbance among working and older age groups: An analysis based on the European Social Survey. International Psychogeriatrics, 23(9), 1413–1420. 21554795 10.1017/S1041610211000664

[jsr14476-bib-0021] Fino, E. , & Mazzetti, M. (2019). Monitoring healthy and disturbed sleep through smartphone applications: A review of experimental evidence. Sleep & Breathing, 23(1), 13–24. 29687190 10.1007/s11325-018-1661-3

[jsr14476-bib-0022] Freeman, D. , Sheaves, B. , Waite, F. , Harvey, A. G. , & Harrison, P. J. (2020). Sleep disturbance and psychiatric disorders. The Lancet Psychiatry, 7(7), 628–637. 32563308 10.1016/S2215-0366(20)30136-X

[jsr14476-bib-0023] Galende‐Sánchez, E. , & Sorman, A. H. (2021). From consultation toward co‐production in science and policy: A critical systematic review of participatory climate and energy initiatives. Energy Research & Social Science, 73, 101907.

[jsr14476-bib-0024] Garbers, S. , Ancheta, A. J. , Gold, M. A. , Maier, M. , & Bruzzese, J.‐M. (2023). Sleeping healthy, living healthy: Using iterative, participatory processes to develop and adapt an integrated sleep hygiene/mind‐body integrative health intervention for urban adolescents. Health Promotion Practice, 25, 15248399231184453. 10.1177/15248399231184453 PMC10808277 37491898

[jsr14476-bib-0025] Harcourt, E. , & Crepaz‐Keay, D. (2023). Co‐production is good, but other things are good too. Royal Institute of Philosophy Supplement, 94, 157–172.

[jsr14476-bib-0026] Hart, R. (1992). Children's participation: From tokenism to citizenship (p. 4). Innocenti Essays.

[jsr14476-bib-0027] Hassan, W. , Zafar, M. , Noreen, H. , Ara, A. , Duarte, A. E. , Kamdem, J. P. , Kamal, M. A. , & da Rocha, J. B. T. (2021). Sleep disorders research from 1945 to 2020: A bibliometric analysis. CNS & Neurological Disorders Drug Targets, 20(7), 574–593. 33602110 10.2174/1871527320666210218085341

[jsr14476-bib-0028] Ibáñez, V. , Silva, J. , & Cauli, O. (2018). A survey on sleep assessment methods. PeerJ, 6, e4849. 29844990 10.7717/peerj.4849 PMC5971842

[jsr14476-bib-0029] Jakobsson, C. E. , Genovesi, E. , Afolayan, A. , Bella‐Awusah, T. , Omobowale, O. , Buyanga, M. , Kakuma, R. , & Ryan, G. K. (2023). Co‐producing research on psychosis: A scoping review on barriers, facilitators and outcomes. International Journal of Mental Health Systems, 17(1), 25. 37644476 10.1186/s13033-023-00594-7 PMC10466887

[jsr14476-bib-0030] Jehan, S. , Myers, A. K. , Zizi, F. , Pandi‐Perumal, S. R. , Jean‐Louis, G. , Singh, N. , Ray, J. , & Samy, M. F., I . (2018). Sleep health disparity: The putative role of race, ethnicity and socioeconomic status. Sleep Medicine Disorder, 2(5), 127–133. PMC6553614 31179440

[jsr14476-bib-0031] Lemoine, P. , Ebert, D. , Koga, Y. , & Bertin, C. (2022). Public interest and awareness regarding general health, sleep quality and mental wellbeing during the early COVID‐19 pandemic period: An exploration using Google trends. Sleep Epidemiology, 2, 100017. 35673330 10.1016/j.sleepe.2021.100017 PMC8604793

[jsr14476-bib-0032] Lim, D. C. , Najafi, A. , Afifi, L. , Bassetti, C. , Buysse, D. J. , Han, F. , Högl, B. , Melaku, Y. A. , Morin, C. M. , Pack, A. I. , Poyares, D. , Somers, V. K. , Eastwood, P. R. , Zee, P. C. , Jackson, C. L. , & World Sleep Society Global Sleep Health Taskforce . (2023). The need to promote sleep health in public health agendas across the globe. The Lancet Public Health, 8(10), e820–e826. 37777291 10.1016/S2468-2667(23)00182-2 PMC10664020

[jsr14476-bib-0033] Lyall, L. M. , Sangha, N. , Wyse, C. , Hindle, E. , Haughton, D. , Campbell, K. , Brown, J. , Moore, L. , Simpson, S. A. , Inchley, J. C. , & Smith, D. J. (2020). Accelerometry‐assessed sleep duration and timing in late childhood and adolescence in Scottish schoolchildren: A feasibility study. PLoS One, 15(12), e0242080. 33259503 10.1371/journal.pone.0242080 PMC7707491

[jsr14476-bib-0034] Malterud, K. , Siersma, V. D. , & Guassora, A. D. (2016). Sample size in qualitative interview studies: Guided by information Power. Qualitative Health Research, 26(13), 1753–1760. 26613970 10.1177/1049732315617444

[jsr14476-bib-0035] McConachie, H. , Livingstone, N. , Morris, C. , Beresford, B. , le Couteur, A. , Gringras, P. , Garland, D. , Jones, G. , Macdonald, G. , Williams, K. , & Parr, J. R. (2018). Parents suggest which indicators of progress and outcomes should be measured in young children with autism spectrum disorder. Journal of Autism & Developmental Disorders, 48(4), 1041–1051. 28861649 10.1007/s10803-017-3282-2 PMC5861173

[jsr14476-bib-0036] Murray, A. L. , & Xie, T. (2024). Engaging adolescents in contemporary longitudinal health research: Strategies for promoting participation and retention. Journal of Adolescent Health, 74(1), 9–17. 10.1016/j.jadohealth.2023.06.032 37690009

[jsr14476-bib-0037] Namazi, S. , Dugan, A. G. , Cavallari, J. M. , Rinker, R. D. , Preston, J. C. , Steele, V. L. , el Ghaziri, M. , & Cherniack, M. G. (2023). Participatory design of a sleep intervention with correctional supervisors using a root causes approach. American Journal of Industrial Medicine, 66(2), 167–177. 36537998 10.1002/ajim.23452

[jsr14476-bib-0038] Norton, M. J. (2021). Co‐production within child and adolescent mental health: A systematic review. International Journal of Environmental Research and Public Health, 18(22), 11897. 34831653 10.3390/ijerph182211897 PMC8623106

[jsr14476-bib-0039] Pais, M. S. , & Bissell, S. (2006). Overview and implementation of the UN convention on the rights of the child. Lancet, 367(9511), 689–690. 16503467 10.1016/S0140-6736(06)68267-6

[jsr14476-bib-0040] Perez‐Pozuelo, I. , Zhai, B. , Palotti, J. , Mall, R. , Aupetit, M. , Garcia‐Gomez, J. M. , Taheri, S. , Guan, Y. , & Fernandez‐Luque, L. (2020). The future of sleep health: A data‐driven revolution in sleep science and medicine. npj Digital Medicine, 3(1), 42. 32219183 10.1038/s41746-020-0244-4 PMC7089984

[jsr14476-bib-0041] Perkes, S. J. , Huntriss, B. , Skinner, N. , Leece, B. , Dobson, R. , Mattes, J. , Hall, K. , & Bonevski, B. (2022). Development of a maternal and child mHealth intervention with aboriginal and Torres Strait islander mothers: Co‐design approach. JMIR Formative Research, 6(7), e33541. 35802404 10.2196/33541 PMC9308065

[jsr14476-bib-0042] Pettican, A. , Goodman, B. , Bryant, W. , Beresford, P. , Freeman, P. , Gladwell, V. , Kilbride, C. , & Speed, E. (2023). Doing together: Reflections on facilitating the co‐production of participatory action research with marginalised populations. Qualitative Research in Sport, Exercise and Health, 15(2), 202–219.

[jsr14476-bib-0043] Power, L. , Murray, A. L. , Hoxha, D. , Xie, T. , & Bartlett, T. (2024). Co‐producing an ecological momentary assessment measurement burst mental health study with young people: The MHIM co‐production protocol. PsyArXiv. 10.1111/hex.70218 PMC11894036 40065653

[jsr14476-bib-0044] Ramar, K. , Malhotra, R. K. , Carden, K. A. , Martin, J. L. , Abbasi‐Feinberg, F. , Aurora, R. N. , Kapur, V. K. , Olson, E. J. , Rosen, C. L. , Rowley, J. A. , Shelgikar, A. V. , & Trotti, L. M. (2021). Sleep is essential to health: An American Academy of Sleep Medicine position statement. Journal of Clinical Sleep Medicine, 17(10), 2115–2119. 34170250 10.5664/jcsm.9476 PMC8494094

[jsr14476-bib-0045] Rapaport, P. , Webster, L. , Horsley, R. , Kyle, S. D. , Kinnunen, K. M. , Hallam, B. , Pickett, J. , Cooper, C. , Espie, C. A. , & Livingston, G. (2018). An intervention to improve sleep for people living with dementia: Reflections on the development and co‐production of DREAMS:START (Dementia RElAted Manual for sleep: STrAtegies for RelaTives). Dementia (London, England), 17(8), 976–989. 30373463 10.1177/1471301218789559

[jsr14476-bib-0046] Ryan, A. T. , Brenner, L. A. , Ulmer, C. S. , Mackintosh, M. A. , & Greene, C. J. (2023). The use of evaluation panels during the development of a digital intervention for veterans based on cognitive behavioral therapy for insomnia: Qualitative evaluation study. JMIR Formative Research, 7(101726394), e40104. 36877553 10.2196/40104 PMC10028512

[jsr14476-bib-0047] Scott, H. , Lack, L. , & Lovato, N. (2020). A systematic review of the accuracy of sleep wearable devices for estimating sleep onset. Sleep Medicine Reviews, 49, 101227. 31901524 10.1016/j.smrv.2019.101227

[jsr14476-bib-0048] Shaw, A. , do, T. N. T. , Harrison, L. , Marczak, M. , Dimitriou, D. , & Joyce, A. (2022). Sleep and cognition in people with autism spectrum condition: A systematic literature review. Review Journal of Autism and Developmental Disorders, 9(3), 416–426.

[jsr14476-bib-0049] Smith, B. , Williams, O. , Bone, L. , & Moving Social Work Co‐production Collective . (2023). Co‐production: A resource to guide co‐producing research in the sport, exercise, and health sciences. Qualitative Research in Sport, Exercise and Health, 15(2), 159–187.

[jsr14476-bib-0050] Smith, H. , Budworth, L. , Grindey, C. , Hague, I. , Hamer, N. , Kislov, R. , van der Graaf, P. , & Langley, J. (2022). Co‐production practice and future research priorities in United Kingdom‐funded applied health research: A scoping review. Health Research Policy and Systems, 20(1), 36. 35366898 10.1186/s12961-022-00838-x PMC8976994

[jsr14476-bib-0051] Smith, M. T. , McCrae, C. S. , Cheung, J. , Martin, J. L. , Harrod, C. G. , Heald, J. L. , & Carden, K. A. (2018). Use of actigraphy for the evaluation of sleep disorders and circadian rhythm sleep‐wake disorders: An American Academy of sleep medicine systematic review, meta‐analysis, and GRADE assessment. Journal of Clinical Sleep Medicine, 14(7), 1209–1230. 29991438 10.5664/jcsm.7228 PMC6040804

[jsr14476-bib-0052] Staniszewska, S. , Brett, J. , Simera, I. , Seers, K. , Mockford, C. , Goodlad, S. , Altman, D. G. , Moher, D. , Barber, R. , Denegri, S. , Entwistle, A. , Littlejohns, P. , Morris, C. , Suleman, R. , Thomas, V. , & Tysall, C. (2017). GRIPP2 reporting checklists: Tools to improve reporting of patient and public involvement in research. BMJ, 358, j3453. 28768629 10.1136/bmj.j3453 PMC5539518

[jsr14476-bib-0053] Stephanie, H. Y. , Gumport, N. B. , Mirzadegan, I. A. , Mei, Y.‐J. , Hein, K. , & Harvey, A. G. (2020). Addressing the challenges of recruitment and retention in sleep and circadian clinical trials. Behavioral Sleep Medicine 18(1), 23–34. 31030562 10.1080/15402002.2018.1518230 PMC6819244

[jsr14476-bib-0054] Tisdall, E. K. M. (2017). Conceptualising children and young people's participation: Examining vulnerability, social accountability and co‐production. The International Journal of Human Rights, 21(1), 59–75.

[jsr14476-bib-0055] Tricco, A. C. , Lillie, E. , Zarin, W. , O'Brien, K. K. , Colquhoun, H. , Levac, D. , Moher, D. , Peters, M. D. J. , Horsley, T. , Weeks, L. , Hempel, S. , Akl, E. A. , Chang, C. , McGowan, J. , Stewart, L. , Hartling, L. , Aldcroft, A. , Wilson, M. G. , Garritty, C. , … Straus, S. E. (2018). PRISMA extension for scoping reviews (PRISMA‐ScR): Checklist and explanation. Annals of Internal Medicine, 169(7), 467–473. 30178033 10.7326/M18-0850

[jsr14476-bib-0056] Vandendriessche, A. , Deforche, B. , Dhondt, K. , Altenburg, T. M. , & Verloigne, M. (2023). Combining participatory action research with intervention mapping to develop and plan the implementation and evaluation of a healthy sleep intervention for adolescents. Health Promotion Perspectives, 13(4), 316–329. 38235009 10.34172/hpp.2023.37 PMC10790120

[jsr14476-bib-0057] Vargas, C. , Whelan, J. , Brimblecombe, J. , & Allender, S. (2022). Co‐creation, co‐design, co‐production for public health ‐ a perspective on definition and distinctions. Public Health Research and Practice, 32(2). 10.17061/phrp3222211 35702744

[jsr14476-bib-0058] Vera San Juan, N. , Oram, S. , Pinfold, V. , Temple, R. , Foye, U. , Simpson, A. , Johnson, S. , Hardt, S. , Abdinasir, K. , & Edbrooke‐Childs, J. (2022). Priorities for future research about screen use and adolescent mental health: A participatory prioritization study. Frontiers in Psychiatry, 13, 697346. 35599756 10.3389/fpsyt.2022.697346 PMC9120839

[jsr14476-bib-0059] von Köppen, M. , Kümpers, S. , & Hahn, D. (2022). Co‐production of knowledge and dialogue: A reflective analysis of the space between academic and lay co‐researchers in the early stages of the research process. Forum Qualitative Sozialforschung/Forum: Qualitative Social Research, 23(1).

[jsr14476-bib-0060] Withers, A. , Maul, J. , Rosenheim, E. , O'Donnell, A. , Wilson, A. , & Stick, S. (2022). Comparison of home ambulatory type 2 polysomnography with a portable monitoring device and in‐laboratory type 1 polysomnography for the diagnosis of obstructive sleep apnea in children. Journal of Clinical Sleep Medicine, 18(2), 393–402. 34323688 10.5664/jcsm.9576 PMC8804994

[jsr14476-bib-0061] Yoon, H. , & Choi, S. H. (2023). Technologies for sleep monitoring at home: Wearables and nearables. Biomedical Engineering Letters, 13(3), 313–327. 37519880 10.1007/s13534-023-00305-8 PMC10382403

